# The importance of language

**DOI:** 10.1093/jhps/hny002

**Published:** 2018-02-05

**Authors:** Richard (Ricky) Villar

**Affiliations:** Editor-in-Chief, Journal of Hip Preservation Surgery

There is a problem with being English and that, you see, is my language. Somehow it has become the *lingua franca* of science. Strange when one considers that 1197 million speak Chinese, 399 million Spanish and English can only boast 335 million. It is the third largest language in the world [1]. But the number of people speaking is one thing; where it is spoken is another—101 countries and territories worldwide, in 94 of which English is the official language. And if you count those for whom English is a second language, then 335 million becomes 1 billion in an instant. English must not relax, however, as French is on its tail. By 2050, 750 million will be Francophones [2], surpassing English in an instant.

To submit a paper in well-constructed English is clearly of paramount importance, a requirement that is unlikely to disappear. The first shot is the best shot and many, in a host of specialties, have looked at the use of English and its influence on acceptance, influence, outcome and impact. Although actual knowledge of English is not always critical, the use of third-party language editing is an advantage, as is the assistance of an advisor with a high h-index [3]. Journals have discovered that a larger share of English-language articles in multi-language medical journals is associated with greater international recognition and fewer self-citations [4].

But although English is widely employed in the scientific literature, some studies have shown up to 35.6% of scientific publications may not be in the English language [5]. The problem is, citation frequency appears integrally linked to the language used, papers in English having a 6- or 7-fold higher chance of citation than papers in German or French [6]. If you decide to submit in Spanish to a Spanish journal, and the journal turns you down, work has shown there is a lesser chance of another publication accepting you [7]. Some have even recommended that non-English language journals change their publication language, or at least adopt a bilingual approach, as English language is associated with a higher impact factor [8], that key metric that we all love to hate.

The appearance of key publications in a non-English journal has on occasion been dangerous. Remember H5N1, bird ‘flu? Chinese researchers reported it in January 2004 [9] based on their findings in south-eastern China in 2003. The English-speaking world thought not to look and it took until August 2004, when the paper’s authors had presented their findings at an international symposium in Beijing that the word was out. Only then did the World Health Organisation and UN Food and Agriculture Organisation swing into action. All because the original paper had been in Chinese and no one had done their reading.

Of course, these observations do not apply to *JHPS* as the journal is in English, and yet I am constantly impressed at the tremendous linguistic abilities among our non-English authors. Truth be known, there are a few authors from non-English lands whose English is better than a native Brit. Some of our non-English authors follow language by the book.

English or non-English, what struck me as interesting in our last issue, issue 4.4? Well everything, of course, otherwise we would never have published. However, the treatment algorithm for ischiofemoral impingement by Gollwitzer *et al*. [10] was certainly fascinating, especially with my impression that we are diagnosing this entity more frequently than a few years ago. Meanwhile it was good to learn that the longer one dallied before undertaking surgery for FAI, the less likely one was to have a satisfactory result. Thank you, Dierckman *et al.* [11] for that one. By all means wait and see, but do not wait for ever.

And for this issue, issue 5.1? Again, I am spoiled for choice but I have much enjoyed the increasing contributions from our open surgery colleagues. Papers are coming in from them quick and fast. So, no wonder I was held spellbound by the Case Report from Sloan and Kamath [12], who used dermal allograft in a Colonna arthroplasty when managing a chronic dislocation. And how about the detailed debate offered by Thanacharoenpanich *et al.* [13] as to whether arthroscopy or arthrotomy is required at a periacetabular osteotomy? They appear to leave the way open for both.

So, as ever, please enjoy this issue of *JHPS*. It is published for you, the hip preservation practitioner, and is filled from cover to cover with brilliance. I commend this issue to you in its entirety.

My very best wishes to you all.
